# Prevalence and clinical significance of anatomic variant in cephalic arch on preoperative mapping venography

**DOI:** 10.1038/s41598-022-22372-0

**Published:** 2022-10-17

**Authors:** Hyoung Nam Lee, Seung Boo Yang, Woong Hee Lee, Youngjong Cho, Sung-Joon Park, Sangjoon Lee

**Affiliations:** 1 Department of Radiology, Soonchunhyang University College of Medicine, Cheonan Hospital, Cheonan, Korea; 2 Department of Radiology, Soonchunhyang University College of Medicine, Gumi Hospital, Gumi, Korea; 3 Department of Radiology, University of Ulsan College of Medicine, Gangneung Asan Hospital, Gangneung, Korea; 4 Department of Radiology, Korea University College of Medicine, Korea University Ansan Hospital, Ansan, Korea; 5 Vascular Center, The Eutteum Orthopedic Surgery Hospital, Paju, Korea

**Keywords:** Anatomy, Medical imaging, Risk factors, Renal replacement therapy

## Abstract

The aim of the current study was to determine the prevalence of anatomic variant in cephalic arch on preoperative mapping venography and evaluate patency rates and predictors of patency in patients with brachiocephalic fistulas. The prevalence of anatomic variant in cephalic arch was retrospectively evaluated in 1004 consecutive patients who underwent bilateral preoperative mapping venography from July 2006 to December 2018 in a single center. The overall prevalence of anatomic variant in cephalic arch was 17.2% (173/1004). For patency analysis, 128 patients with brachiocephalic fistulas were divided into two groups: a standard anatomy (SA) group (*n* = 97) and a variant anatomy (VA) group (*n* = 31). There were no significant differences in clinical characteristics between the two groups. The primary patency rate did not differ significantly between the two groups. The secondary patency rate was significantly (*p* = 0.009) lower in the VA group than in the SA group. Older age (HR 1.03; 95% CI 1.01–1.05; *p* = 0.007) was a negative predictor of primary patency, and antiplatelet agent (HR 0.53; 95% CI 0.33–0.84; *p* = 0.007) and large-diameter cephalic vein (HR 0.52; 95% CI 0.31–0.86; *p* = 0.012) were positive predictors of primary patency. Older age (HR 1.04; 95% CI 1.01–1.07; *p* = 0.011) and anatomic variant in cephalic arch (HR 2.9; 95% CI 1.19–7.06; *p* = 0.019) were negative predictors of secondary patency. The current study provides insight into the clinical significance of anatomic variant in cephalic arch. Anatomic variant in cephalic arch should be considered as a potential risk factor for decreased patency of brachiocephalic fistula during preoperative planning.

## Introduction

Preoperative vascular mapping for hemodialysis access placement has been increasingly adopted to evaluate the precise vascular anatomy and to improve dialysis result by proper selection of target vessel^1–3^. Doppler ultrasound can provide an accurate assessment of luminal diameter with a flow velocity. It is preferred in patients with minimal residual renal function^4^. Venography is extremely useful for evaluating central vein stenosis and provide a road map of entire venous anatomy^5^. Brachiocephalic fistula is usually placed in a patient with a forearm fistula that has failed or a patient whose forearm vessels are unsuitable for fistula formation on preoperative vascular mapping^6,7^.

Cephalic arch is one of the most important anatomic landmarks of brachiocephalic fistula. It is the final bridge of the cephalic vein as it arcs through the deltopectoral groove to join the axillary vein^8^. Careful preoperative assessment of the cephalic arch is required because it is a unique venous outflow tract and a frequent site of stenosis in brachiocephalic fistula^9–11^. On preoperative mapping venography, anatomic variant in cephalic arch has been reported to be as high as 8.7% in published literature^1,12^. However, there is paucity of data on the clinical significance of anatomic variant in cephalic arch^13^. Thus, the aim of the current study was to determine the prevalence of anatomic variant in cephalic arch on preoperative mapping venography and to evaluate patency rates and predictors of patency in patients with brachiocephalic fistulas.

## Methods

The Institutional Review Board of Soonchunhyang University Cheonan Hospital (IRB No. 2019-12-005) approved this retrospective single-center study and waived the requirement of written informed consent for use of clinical and imaging data. All methods were carried out in accordance with relevant guidelines and regulations.

### Venography protocol

During the study period, mapping venography was performed as a routine imaging procedure before access creation. Intravenous accesses were placed in both hands with 20-guage indwelling needles. Venography was performed using a 50:50 dilution mixture (20–30 ml) of nonionic low-osmolar contrast media (Omnipaque 350; GE Healthcare, Jupiter, Florida, USA) with normal saline. A tourniquet was applied to the level of the upper arm near the axilla^14^. After veins were filled with contrast media, digital spot images were taken. Evaluations of the cephalic arch and the central vein were achieved with contrast media injection as an untied tourniquet at the same time.

### Operative technique

All surgical procedures were performed by one of the two vascular surgeons (10 and 5 years of experience, respectively). Brachiocephalic fistulas were placed in patients whose preoperative mapping indicated brachial artery and cephalic vein in the antecubital space. The minimum cephalic vein diameter required was set at 2 mm. If possible, the non-dominant side was used for construction of the fistula. Under local anesthesia, a transverse skin incision was made at about 1 cm above the elbow crease. The bicipital fascia was incised, and the brachial artery was exposed. The artery was incised after clamping, an end-to-side vein-to artery anastomosis was performed with a running 7–0 polypropylene suture with a limited arteriotomy. Subsequently, clamps were removed and bleeding was controlled. The fistula could then be easily palpated through the skin.

### Study design

**Figure 1:**
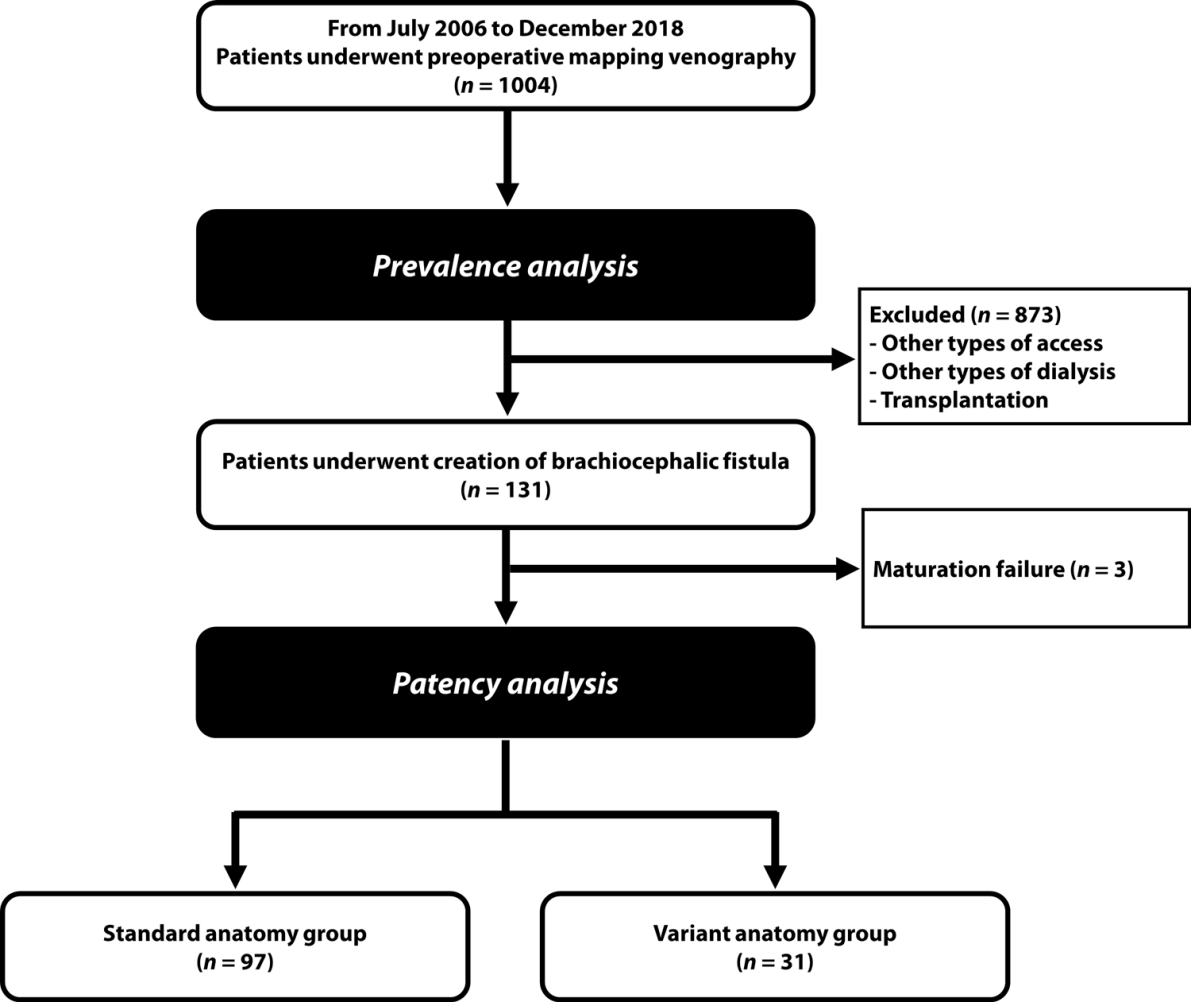
Study flow diagram.

The prevalence of anatomic variant in cephalic arch was evaluated in 1004 consecutive patients who underwent bilateral preoperative mapping venography from July 2006 to December 2018. All venograms were reviewed by two interventional radiologists (10 and 5 years of experience, respectively) who were blinded for patient characteristics to minimize potential bias. Of 1004 patients, 131 underwent creation of brachiocephalic fistula. Three patients who failed to have a mature fistula were excluded. A total of 128 patients (age, 60.71 ± 13.3 years; men, 53.1%) were eligible for patency analysis. They were divided into two groups: a standard anatomy (SA) group (*n* = 97) and a variant anatomy (VA) group (*n* = 31). The data collection regarding patency and interventions of each patient was based on patient medical records. The study flow diagram is depicted in Fig. 1.

**Figure 2:**
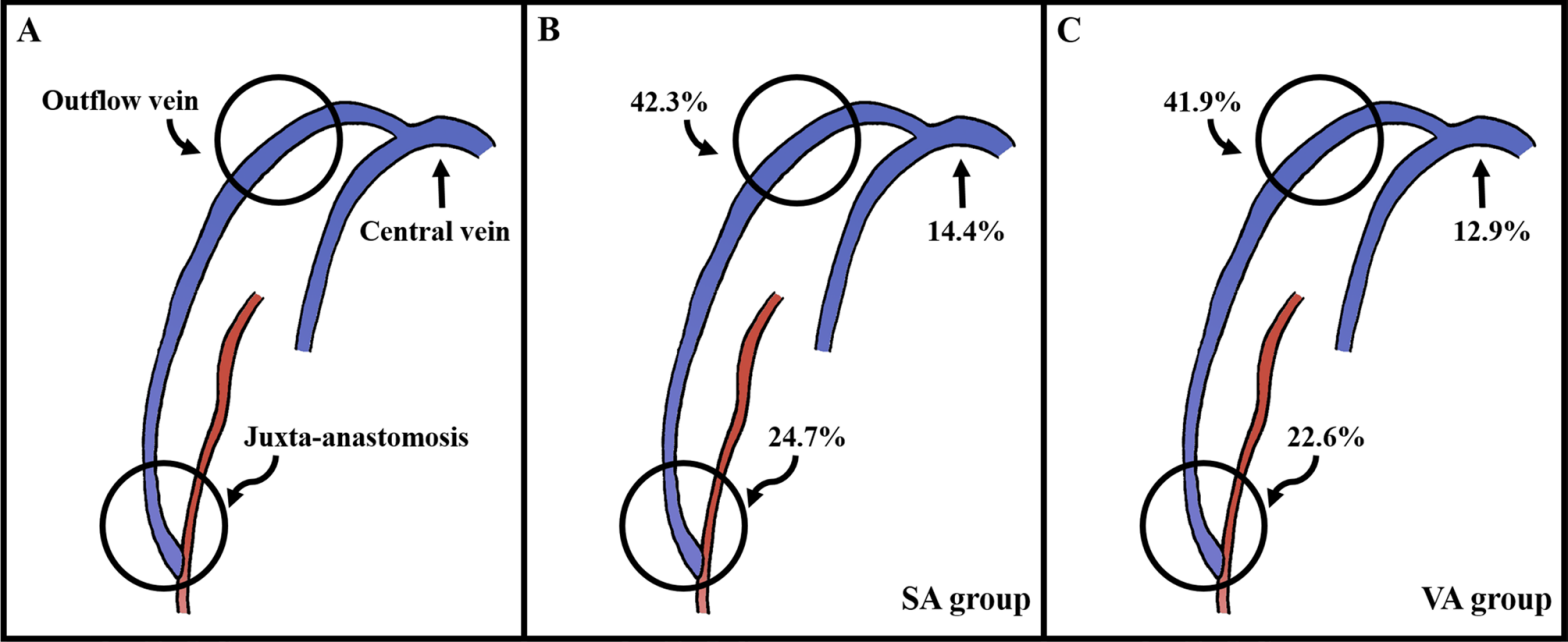
Sites of stenosis in brachiocephalic fistula. Illustrations demonstrate (2**A**) classification of sites of stenosis, (2**B**) sites of stenosis in the SA group, and (2**C**) sites of stenosis in the VA group. SA: standard anatomy; VA: variant anatomy.

Primary endpoints included primary and secondary patency rates defined based on Reporting Standards of the Society for Vascular Surgery and the American Association for Vascular Surgery^15^. Primary patency was defined as the interval from the time of access placement until any intervention designed to maintain or reestablish patency, access thrombosis. Secondary patency was defined as the interval from the time of access placement until access abandonment including endovascular interventions. Secondary endpoints included the number of intervention and the location of stenosis requiring intervention. The location of stenosis requiring intervention was categorized anatomically into juxta-anastomosis stenosis (< 3 cm from anastomosis), outflow vein (cephalic arch) stenosis, or central vein stenosis (Fig. 2).

### Statistical analysis

For descriptive statistics, continuous variables are presented as mean ± standard deviation and categorical variables are presented as absolute and relative frequencies. For comparative analysis, the Wilcoxon rank-sum test was used for continuous variables and the Chi-squared test or Fisher’s exact test was used for categorical variables. The Kaplan–Meier technique and the log-rank test were used to evaluate and compare access patency rates between the two groups. Censoring of endpoints occurred in the setting of patient death with a functioning access, renal transplantation with a functioning access, and functioning access at the time of the last documented follow-up. Potential predictors of access patency were analyzed with a Cox proportional-hazards model. Variables with a *p*-value of less than 0.1 on univariable analysis were included in the multivariable analysis. A *p*-value < 0.05 was considered statistically significant. All statistical analyses were executed using R version 3.6.3 software (Foundation for Statistical Computing, Vienna, Austria).

## Results

**Figure 3:**
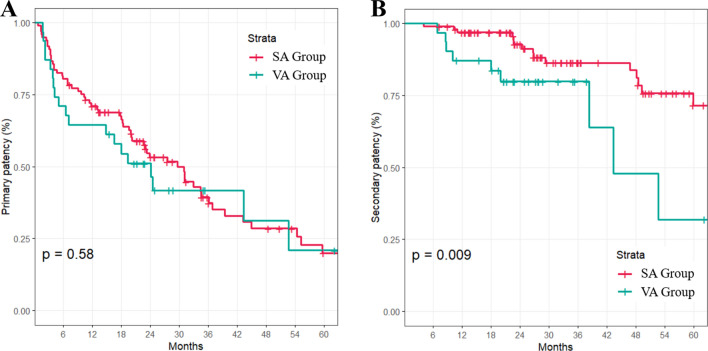
Kaplan–Meier survival analysis. Kaplan–Meier curves demonstrate (3**A**) primary and (3**B**) secondary patency rates in SA and VA groups. SA: standard anatomy; VA: variant anatomy.

The overall prevalence of anatomic variant in cephalic arch was 17.2% (173/1004). For patency analysis, 128 patients with brachiocephalic fistulas were divided into two groups: the SA group (*n* = 97) and the VA group (*n* = 31). There were no significant differences in clinical characteristics between the two groups (Table 1). The mean follow-up duration was 36.5 months for the SA group and 29.7 months for the VA group, showing no significant difference between the two groups (*p* = 0.215). The primary patency rate did not differ significantly between the two groups either (*p* = 0.58) (Fig. 3). However, the secondary patency rate was significantly lower in the VA group than in the SA group (*p* = 0.009). The mean number of intervention required was 1.07 ± 1.32 times in the SA group and 1.06 ± 1.69 in the VA group, showing no significant difference (*p* = 0.607). There was no significant difference in the location of stenosis between the two groups either: juxta-anastomosis, outflow vein, or central vein (*p* > 0.99) (Fig. 2).

**Table 1:** Comparison of clinical characteristics between the two groups.

Characteristics	All (*n* = 128)	SA (*n* = 97)	VA (*n* = 31)	*p*-value
Age, years	60.71 ± 13.3	59.95 ± 13.2	63.1 ± 13.56	0.299
Male	68 (53.12%)	53 (54.64%)	15 (48.39%)	0.689
Smoking	25 (19.53%)	21 (21.65%)	4 (12.9%)	0.419
CVC placement	23 (17.97%)	16 (16.49%)	7 (22.58%)	0.617
MAP, mmHg	96.48 ± 11.88	96.05 ± 12.26	97.85 ± 10.66	0.546
BMI, kg/m^2^	24.28 ± 3.85	24.29 ± 4.01	24.23 ± 3.38	0.775
eGFR, mL/min/1.73 m^2^	10.65 ± 3.73	10.64 ± 3.57	10.69 ± 4.24	0.956
Cephalic vein, mm	2.49 ± 0.51	2.53 ± 0.53	2.36 ± 0.43	0.112
Medication	92 (71.88%)	67 (69.07%)	25 (80.65%)	0.309
Antiplatelet agent	88 (68.75%)	64 (65.98%)	24 (77.42%)	0.33
Anticoagulation agent	4 (3.12%)	3 (3.09%)	1 (3.23%)	> 0.99
≥ 2 Comorbidities	95 (74.22%)	74 (76.29%)	21 (67.74%)	0.477
Hypertension	114 (95%)	92 (94.85%)	22 (95.65%)	> 0.99
Diabetes	84 (70%)	69 (71.13%)	15 (65.22%)	0.761
Cerebrovascular disease	24 (20%)	22 (22.68%)	2 (8.7%)	0.158
Cardiovascular disease	28 (23.33%)	23 (23.71%)	5 (21.74%)	> 0.99
PAOD	7 (5.83%)	4 (4.12%)	3 (13.04%)	0.128

SA, standard anatomy; VA, variant anatomy; CVC, central vein catheterization; MAP, mean arterial pressure; BMI, body mass index; eGFR, estimated glomerular filtration rate; PAOD, peripheral arterial occlusive disease.

Results of univariable and multivariable Cox proportional hazards regression analysis to identify predictors associated with access patency are summarized in Tables 2 and 3. Multivariable analysis indicated that older age (HR 1.03; 95% CI 1.01–1.05; *p* = 0.007) was a negative predictor of primary patency, and antiplatelet agent (HR 0.53; 95% CI 0.33–0.84; *p* = 0.007) and large-diameter cephalic vein (HR 0.52; 95% CI 0.31–0.86; *p* = 0.012) were positive predictors of primary patency. Older age (HR 1.04; 95% CI 1.01–1.07; *p* = 0.011) and anatomic variant in cephalic arch (HR 2.9; 95% CI 1.19–7.06; *p* = 0.019) were negative predictors of secondary patency. There was no multicollinearity because variance inflation factors were less than the threshold value of 2.

**Table 2:** Predictors of primary patency.

	Univariable analysis			Multivariable analysis		
Variables	HR	95% CI	*p*-value	HR	95% CI	*p*-value
Age	1.027	1.009–1.045	0.003*	1.026	1.007–1.045	0.007*
Male	0.921	0.592–1.435	0.717			
Smoking	0.746	0.411–1.354	0.336			
CVC placement	1.196	0.678–2.112	0.537			
MAP, mmHg	0.99	0.972–1.008	0.264			
BMI, kg/m^2^	0.997	0.94–1.058	0.919			
eGFR, mL/min/1.73m²	1.02	0.958–1.085	0.537			
Cephalic vein, mm	0.54	0.326–0.895	0.017*	0.515	0.308–0.862	0.012*
Antiplatelet	0.569	0.361–0.896	0.015*	0.528	0.333–0.837	0.007*
Anticoagulation	1.651	0.518–5.264	0.397			
≥ 2 Comorbidities	1.099	0.655–1.841	0.722			
Anatomical variant	1.157	0.688–1.943	0.583			

CVC, central vein catheterization; MAP, mean arterial pressure; BMI, body mass index; eGFR, estimated glomerular filtration rate.

*p < 0.05 means statistical significance.

**Table 3:** Predictors of secondary patency.

	Univariable analysis			Multivariable analysis		
Variables	HR	95% CI	*p*-value	HR	95% CI	*p*-value
Age	1.038	1.006–1.07	0.019*	1.04	1.008–1.073	0.011*
Male sex	0.785	0.358–1.722	0.545			
Smoking	0.903	0.309–2.639	0.852			
CVC placement	0.982	0.335–2.878	0.974			
MAP, mmHg	0.981	0.951–1.012	0.229			
BMI, kg/m^2^	1.021	0.919–1.135	0.695			
eGFR, mL/min/1.73m²	1.067	0.961–1.184	0.224			
Cephalic vein, mm	0.423	0.154–1.164	0.096	0.533	0.196–1.453	0.219
Antiplatelet	0.959	0.413–2.224	0.921			
Anticoagulation	1.104	0.148–8.243	0.923			
≥ 2 Comorbidities	0.917	0.381–2.203	0.846			
Anatomical variant	2.923	1.256–6.799	0.013*	2.898	1.189–7.063	0.019*

CVC, central vein catheterization; MAP, mean arterial pressure; BMI, body mass index; eGFR, estimated glomerular filtration rate.

*p < 0.05 means statistical significance.

## Discussion

Anatomic variants of upper arm veins are frequently encountered on preoperative mapping venography. Their common locations include brachial-basilic confluence and cephalic arch^1,12^. Early confluence of basilic and unpaired brachial veins has been described as potential prohibition of basilic vein transposition that can lead to failure of subsequent graft placement^16,17^. On the other hand, the clinical significance of anatomic variant in cephalic arch has not been established yet^13^. In the current study, the association between patency of brachiocephalic fistula and anatomic variant in cephalic arch was investigated. The secondary patency of the VA group was significantly lower than that of the SA group despite the two groups had comparable preoperative clinical characteristics. Anatomic variant in cephalic arch was a significant negative predictor of secondary patency on multivariable analysis.

Anatomic variant in cephalic arch was found in 17.2% of the study population, which was higher than that in previous reports (4–8.7%)^1,12^. These differences may be explained by different indications of mapping venography. The prevalences of the anatomic variant in previous studies were from a select group of patients which at high risk of central venous stenosis, which is not a true prevalence in all patients considered for fistula formation.

In the forearm, radiocephalic fistula drains via basilic, brachial, and cephalic veins. On the other hand, brachiocephalic fistula has a higher flow rate and, in general, drain exclusively via the cephalic vein and its arch^18,19^. Therefore, patency analysis of the current study included only patients with brachiocephalic fistula to assess the clinical significance of anatomic variant in cephalic arch. The cephalic arch is one of the most susceptible site for stenosis in brachiocephalic fistula and the incidence has been reported between 17 and 77%^20–22^. The current study using a cohort of dialysis patients demonstrated that the incidence of cephalic arch stenosis and the primary patency rate was comparable between the two groups, but the secondary patency was not. Older age (HR 1.04; 95% CI 1.01–1.07; *p* = 0.011) and anatomic variant in cephalic arch (HR 2.9; 95% CI 1.19–7.06; *p* = 0.019) were negative predictors of secondary patency. Multivariable analysis showed that anatomic variant in cephalic arch was a negative predictor of secondary patency.

One potential explanation for lower secondary patency in VA group is altered hemodynamics within cephalic arch. A few previous studies focused on underlying hemodynamics in the cephalic arch have been proposed^23,24^. Boghosian et al.^24^ investigated the hemodynamics of anatomic variant in cephalic arch using computational fluid dynamics modeling. They hypothesized that additional channels of cephalic arch could have a protective effect on the development of neointimal hyperplasia by reducing inlet flow rate and wall shear stresses. On the other hand, persistent reduced flow rate within cephalic arch may eventually lead to poor response to interventional treatment and decreased long-term secondary patency.

The current study indicated that age, vein diameter and adjuvant medication were significantly associated with access patency, thus supporting the evidence from several prospective studies and systematic reviews^25–28^. However, controversy remains regarding the benefit of arteriovenous fistula placement in elderly patients and the use of adjuvant medication for prolonging access patency^29–31^. It should also be noted that the current study only targeted patients with brachiocephalic fistula, and caution is required when extrapolating this result to other types of dialysis access.

The current study has certain limitations. The retrospective nature of analysis limited a detailed evaluation of records such as serial follow-up of flow velocity of the fistula. Given the low prevalence of anatomic variant, the small sample size in this single-center cohort might have limited the overall generalizability of study results and subgroup analysis. A larger sample size and a prospectively designed study may be required to validate our results. In addition, there might be missing prognostic factors that affected access patency.

In conclusion, the current study provides insight into the clinical significance of anatomic variant in cephalic arch. Anatomic variant in cephalic arch should be considered as a potential risk factor for decreased patency of brachiocephalic fistula during preoperative planning.

## Data Availability

The datasets used and/or analysed during the current study available from the corresponding author on reasonable request.
